# Cold Water Immersion Enhanced Athletes’ Wellness and 10-m Short Sprint Performance 24-h After a Simulated Mixed Martial Arts Combat

**DOI:** 10.3389/fphys.2018.01542

**Published:** 2018-11-01

**Authors:** Montassar Tabben, Mohammed Ihsan, Nihel Ghoul, Jeremy Coquart, Anis Chaouachi, Helmi Chaabene, Claire Tourny, Karim Chamari

**Affiliations:** ^1^ASPETAR, Orthopaedic and Sports Medicine Hospital, Doha, Qatar; ^2^UFR STAPS, CETAPS, Normandie University – University of Rouen, Rouen, France; ^3^Research Laboratory “Sport Performance Optimization,” National Centre of Medicine and Science in Sport, Tunis, Tunisia; ^4^Sports Performance Research Institute, AUT University, Auckland, New Zealand; ^5^PVF Football Academy, Hang Yen, Vietnam; ^6^Division of Training and Movement Sciences, Research Focus Cognition Sciences, University of Potsdam, Potsdam, Germany; ^7^High Institute of Sports and Physical Education Kef, University of Jendouba, Tunis, Tunisia

**Keywords:** recovery, combat sports, MMA, delayed onset muscle soreness, stress, fatigue

## Abstract

**Objective:** The aim of the present study was to examine the effect of Cold Water Immersion (CWI) on the recovery of physical performance, hematological stress markers and perceived wellness (i.e., Hooper scores) following a simulated Mixed Martial Arts (MMA) competition.

**Methods:** Participants completed two experimental sessions in a counter-balanced order (CWI or passive recovery for control condition: CON), after a simulated MMAs competition (3 × 5-min MMA rounds separated by 1-min of passive rest). During CWI, athletes were required to submerge their bodies, except the trunk, neck and head, in the seated position in a temperature-controlled bath (∼10°C) for 15-min. During CON, athletes were required to be in a seated position for 15-min in same room ambient temperature. Venous blood samples (creatine kinase, cortisol, and testosterone concentrations) were collected at rest (PRE-EX, i.e., before MMAs), immediately following MMAs (POST-EX), immediately following recovery (POST-R) and 24 h post MMAs (POST-24), whilst physical fitness (squat jump, countermovement-jump and 5- and 10-m sprints) and perceptual measures (well-being Hooper index: fatigue, stress, delayed onset muscle soreness (DOMS), and sleep) were collected at PRE-EX, POST-R and POST-24, and at PRE-EX and POST-24, respectively.

**Results:** The main results indicate that POST-R sprint (5- and 10-m) performances were ‘likely to very likely’ (*d* = 0.64 and 0.65) impaired by prior CWI. However, moderate improvements were in 10-m sprint performance were ‘likely’ evident at POST-24 after CWI compared with CON (*d* = 0.53). Additionally, the use of CWI ‘almost certainly’ resulted in a large overall improvement in Hooper scores (*d* = 1.93). Specifically, CWI ‘almost certainly’ resulted in improved sleep quality (*d* = 1.36), stress (*d* = 1.56) and perceived fatigue (*d* = 1.51), and ‘likely’ resulted in a moderate decrease in DOMS (*d* = 0.60).

**Conclusion:** The use of CWI resulted in an enhanced recovery of 10-m sprint performance, as well as improved perceived wellness 24-h following simulated MMA competition.

## Introduction

Mixed Martial Arts (MMA) is characterized by a combination of fighting styles inspired from other combat sports, resulting in an attractive conglomerate of fighting techniques, i.e., strikes, clinch and grappling, and submission (Amtmann et al., 2008; del Vecchio et al., 2011; La Bounty et al., 2011). Following massive media exposure, particularly with the Ultimate Fighting Championship^®^ (UFC) events, its worldwide popularity has grown exponentially, resulting in MMA being one of the most increasingly participated sport at amateur, right through to professional levels (Ghoul et al., 2017). MMA combat is highly intensive in nature, and has previously shown to induce significant fatigue and muscle damage, which was largely persistent throughout a 24-h period. (Amtmann et al., 2008; Ghoul et al., 2017). Results showed that simulated MMA competition induced high HR, blood lactate and RPE (181.3 bpm, 13.6 mmoL, and 6.9 – scale of 10, respectively) (Ghoul et al., 2017). Moreover, a single MMA combat or training session has been shown to increase salivary and blood cortisol levels, as well as urinary neopterin content, indicating profound physiological stress (Ghoul et al., 2017; Lindsay et al., 2017). As such, it is essential that appropriate recovery strategies are undertaken, to ensure that training and/or completion demands are adequately met.

Cold water immersion (CWI) is a recovery modality typically undertaken following endurance (Vaile et al., 2008) and/or team-sport activities (Rowsell et al., 2011). More recently, the use of CWI as a post-exercise recovery strategy has gained considerable popularity within combat sports (Lindsay et al., 2015, 2017). Indeed, CWI seems effective to promote recovery by ameliorating effects of exercise-induced muscle damage (EIMD) and reducing muscle oedema via several mechanisms associated with localized cooling, hydrostatic pressure and the redistribution of blood flow (Ihsan et al., 2016). Moreover, CWI has been purported to confer analgesic effects, which has shown to reduce delayed onset muscle soreness (DOMS), and consequently improve perceptual recovery (Corbett et al., 2011). Recently, there has been increased interest in the efficacy of this recovery modality following MMA-specific activity. For instance, Lindsay et al. (2017) demonstrated reduced inflammation and oxidative stress following an intense MMA training session when CWI was incorporated as a recovery intervention. However, further research investigating the effects of CWI on the recovery of physical performance following simulated combat are warranted. Indeed, given that MMA tournaments are highly demanding in nature, with requirements to compete every 1–2 day(s), the recovery of physical performance should be a crucial outcome measure. Moreover, given that perceptual recovery has been regarded as an important component of recovery, and has shown to be associated with changes in physical performances (Ihsan et al., 2017), it is also imperative to examine whether CWI would potentially be an effective modality to improve perceptual recovery within MMA-specific context. As such, the current study aimed to understand how physiological and perceptual outcomes are simultaneously affected by recovery CWI amongst MMA fighters. The primary purpose of this investigation was to examine the immediate and post 24-h effect of CWI and passive recovery on physical performance following simulated MMA competition. Secondly, we examined changes in the hematological stress markers (creatine kinase, cortisol, and testosterone) associated with recovery. Finally, we studied the perceptual effects of CWI by including wellness measures (i.e., Hooper scores). It was hypothesized that compared to passive recovery, CWI would allow for a better recovery in performance, perceptual, and physiological measures.

## Materials and Methods

### Participants

Twelve male well-trained MMA athletes (mean ± standard deviation for age: 26.5 ± 5.0 years, height: 182.0 ± 7.4 cm, and body mass: 86.2 ± 10.9 kg) participated in this study. All participants competed in local or regional competitions once per month and were regularly training (technical, tactical, and/or physical sessions) 3–4 times per week. Participants were informed about the experimental procedures, potential risks and benefits, and signed an informed consent document before the commencement of the study. All participants were instructed to avoid drinking coffee, tea, cola, or alcohol 24 h preceding the experimental session. All testing procedures were in line with the latest Declaration of Helsinki and the protocol was approved by the institutional IRB-board Ethics committee.

### Experimental Design

One week before the study commencement, the participants undertook a familiarization session where they accustomed to all experimental procedures involved. In a randomized, cross-over manner (repeated measures), participants completed two experimental sessions, conducted 72 h apart. The experimental sessions were conducted at the same time of day (8–10 am), where temperature and humidity were consistent across the sessions (temperature ∼29–30°C; relative humidity ∼38–40%).

**FIGURE 1:**
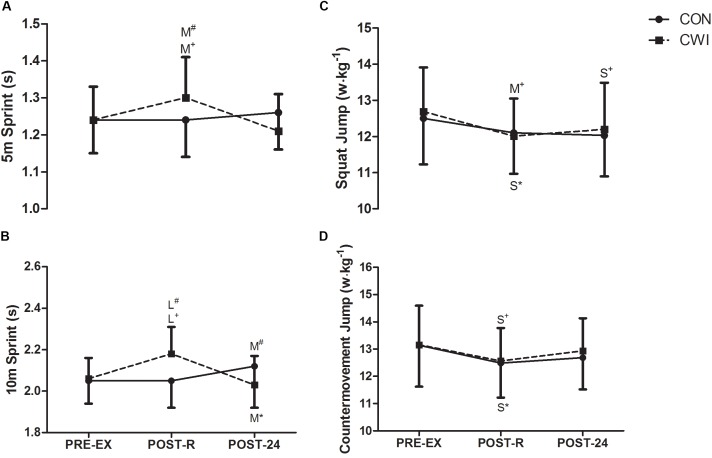
Changes in performances during 5-m sprint **(A)**, 10-m sprint **(B)**, squat jump **(C)** and countermovement jump **(D)** prior to exercise (PRE-EX), immediately after recovery (POST-R) and 24-h following exercise (POST-24) according to recovery modalities: passive recovery (CON) or cold water immersion (CWI). Differences are indicated by the size of the effect (S, small; M, moderate; L, large), accompanied by symbols denoting within (^∗^CON, ^+^CWI) and between group (^#^CON vs. CWI) differences. All reported differences denote ≥75% likelihood that the stated effect is substantially different from the smallest worthwhile change (*d* = 0.2).

The experimental protocol involved four testing time-points (Figure 1), during which physical performance measures and blood sampling was undertaken (described below); (i) prior to simulated MMA combat (MMAs) (PRE-EX), (ii) immediately following MMAs (POST-EX), (iii) immediately following recovery (POST-R), and (iv) 24 h post MMAs (POST-24). Shortly after arriving on day 1, height was assessed to the nearest 0.1 cm using a stadiometer (Holtain Ltd., Crymych, United Kingdom) and body mass was measured to the nearest 0.1 kg using a body composition monitor Tanita BC 545-N (Tanita Health Equipment, Hong Kong, China). Following the completion of a 15-min standardized warm-up and PRE-EX measurements, athletes performed the MMAs protocol. The MMAs consists of 3 × 5-min MMA rounds separated by 1-min of passive rest. They were divided into pairs with a difference of body mass of no more than 10%. All combats were judged by qualified MMA referees according to the official rules. Additionally, as during an official competition, athletes’ coaches were present and they consistently provided guidance, technical advice, and verbal encouragement. No interruption of the combats was permitted even when submissions occurred to ensure the continuity of the fights. Following the completion of the MMAs, POST-EX was undertaken. Three minutes later, athletes performed, one of the following recovery modalities for 15-min in a randomized, counter-balanced manner: CWI or passive recovery (i.e., control modality: CON). Testing measures were immediately undertaken following recovery intervention (i.e., POST-R) and POST-24 was undertaken 24 h following MMAs.

### Recovery Modalities

(1) CWI: Athletes were required to submerge their bodies, except the trunk, neck and head, in the seated position in a temperature-controlled bath (∼10°C) for 15-min. (Lindsay et al., 2017).
(2) Passive recovery (i.e., control modality: CON): Athletes were required to be in a seated position for 15-min with minimal movement at the same room ambient temperature. (Lindsay et al., 2017).

### Experimental Measures

#### Heart Rate Monitoring

Heart rate was continuously recorded using a chest monitor during the sparing bouts and the subsequent rest phase. Recorded data were downloaded on a computer using Polar software (Polar Precision Performance SW5.20, Polar Electro, Kempele, Finland). All irregular heartbeats were automatically identified and replaced with interpolated adjacent R–R interval values with the Polar Software. (Nunan, 2006)

#### Blood Lactate Concentration

Blood lactate concentration was measured at PRE-EX, POST-EX and POST-24. A fingertip capillary blood sample (5 μL) was collected and analyzed for lactate concentration using a calibrated Lactate Pro analyzer (Arkray^®^, Tokyo, Japan).

#### Perceptual Responses

The well-being Hooper index based on ratings relative to (i) fatigue, (ii) stress, (iii) whole-body DOMS, and (iv) sleep has been used as a cost-effective strategy for prevention and early detection of non-functional overreaching/overtraining syndromes (Hooper et al., 1995). The participants were asked to subjectively rate the quality of their prior night-sleep, their fatigue level, stress, and DOMS on a scale of 1–7 before each testing day. 1 was anchored as the positive and 7 the negative end of the continuum for all perceptual variables.

Hooper scores were analyzed as the sum of the four scores as well as for each of the four variables separately. (Hooper et al., 1995).

#### Physical Performances

##### Squat jump (SJ) and countermovement jump (CMJ)

SJ and CMJ performances were undertaken to determine athletes’ explosive power (de Freitas et al., 2017). Jumps were assessed using an infrared jump system (Optojump, Microgate^®^, Bolzano, Italy) interfaced with a microcomputer. Participants were not allowed to use their arms, which were positioned on their hips throughout the jumps. The SJ was completed first followed by the CMJ, with two attempts allowed for each jump type. A 30-s passive recovery was imposed between each jump, which allowed the tester to record vertical jump performance, and to reset the systems for recording of the next trial. A third trial was completed if jump heights varied > 5%. The highest score relative to each type of jump was retained for further analysis.

##### 10-m sprint time

The 10-m sprint assessment was undertaken 2 min following the completion of the jumps tests, and was performed on an outdoor synthetic 20-m sprint court. Participants started in standing position with the toe of the preferred foot positioned forward behind a starting line (i.e., 0.5 m). Time was taken automatically when the athlete voluntarily initiated the test Sprint times were recorded using telemetric photoelectric cells (Racetime 2, Microgate^®^, Bolzano, Italy; accuracy of 0.01 s) placed 0.7 m above the ground. Three attempts were given with 3-min walk-back recovery in-between attempts and the run. The fastest 10-m time (and corresponding 5-m split time) was selected for analysis.

##### Blood sampling and analysis

Venous blood samples (8 mL) were obtained from an antecubital vein in a rested and seated position at PRE-EX, POST-EX, POST-R, and POST-24 to determine serum creatine kinase (CK), cortisol, and testosterone concentrations. Blood was collected into EDTA containing tubes and serum and plasma samples were obtained by centrifuging (3,000 rpm for 15 min at 4°C) and were stored at -80 C° until analysis. All analyses were completed 1 week following the experiment, and performed by a qualified medical personal. All analyzers were calibrated according to the manufacturers’ instructions prior to use. Plasma creatine kinase (CK) activity was used as a marker of muscle damage and was measured spectrophotometrically in an Advia 1800 analyzer (Siemens Healthcare, Erlangen, Germany). Testosterone and cortisol levels were determined by using a solid-phase radio-immunoassay procedure (Immunotech RIA, Beckman Coulter^®^, Brea, United States). The intra-assay coefficients of variation (CVs) for each measurement were established at 3.0% for the CK, 1.6% for Hb and Ht, 2.1% for Cortisol, and 4.3 % for testosterone.

##### Statistical analysis

Data was analyzed using effect sizes and magnitude-based inferences, where within-group and between-group comparisons were undertaken using specifically designed Excel spreadsheets (Hopkins, 2006; Hopkins et al., 2009). These spreadsheets calculated the standardized differences or effect sizes (*d*) with 90% confidence limits (90% CL), and *d* values of 0.2, <0.5, and ≥0.8 were interpreted as small, moderate and large effects, respectively (Cohen, 1988). Magnitude of differences were considered substantial if there was at least a 75% likelihood that the effect was greater than the smallest worthwhile change (i.e., *d* = 0.2), and qualitatively evaluated as 75–<95%: likely; ≥95–99%: very likely; and ≥99%: almost certain. If the chance of substantially higher or lower differences were both >5%, the true difference was assessed as ‘unclear’ (Hopkins et al., 2009).

## Results

The intensity of the simulated combat was consistent across conditions, as HR (CON: 186 ± 15 vs. CWI: 183 ± 15 bpm, *d* = 0.2) and [La^-^] following the combat bouts (CON: 13.3 ± 1.7 vs. CWI: 13.1 ± 1.3 mmol⋅L^-1^, *d* = 0.17) were similar between CON and CWI.

Changes in performance for the 5-m and 10-m sprint, SJ and CMJ are presented in Figure 1. CWI resulted in slower 5-m (*d* = 0.64) and 10-m (*d* = 0.91) sprint time compared with CON at POST-R. In contrast, CWI resulted in improved 10-m sprint performance (*d* = 0.53) at POST-24 compared with CON. Compared with PRE-EX, small to moderate decreases in SJ and CMJ were observed at POST-R both CON and CWI, with no differences (i.e., unclear) between conditions at all time-points (Figure 1C).

**FIGURE 2:**
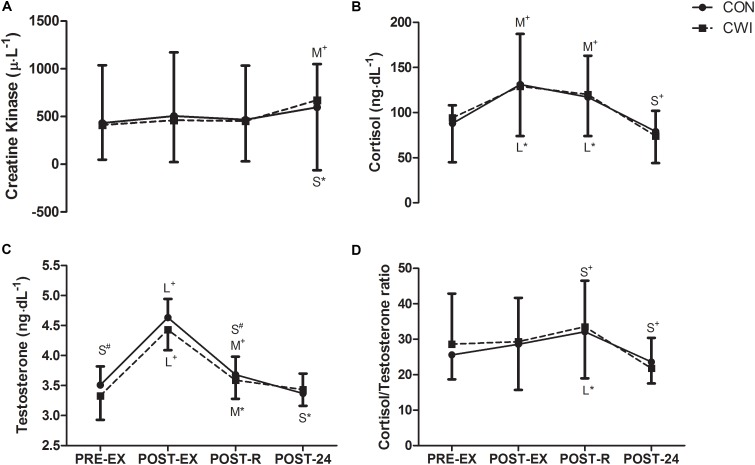
Changes in creatine kinase **(A)**, testosterone **(B)**, cortisol **(C)**, and cortisol/testosterone ratio **(D)** PRE-EX, immediately after exercise (POST-EX), immediately after recovery (POST-R) and 24-h following exercise (POST-24) according to recovery modalities: passive recovery (CON) or CWI. Differences are indicated by the size of the effect (S, small; M, moderate; L, large), accompanied by symbols denoting within (^∗^CON, ^+^CWI) and between group (^#^CON vs. CWI) differences. All reported differences denote ≥75% likelihood that the stated effect is substantially different from the smallest worthwhile change (*d* = 0.2).

Changes in CK, cortisol, testosterone and cortisol/testosterone ratio are presented in Figure 2. The simulated combat resulted in small to large increases in cortisol, testosterone and cortisol:testosterone ratio during the POST-EX and POST-R periods, which receded to levels similar to PRE-EX by POST-24 in both conditions. In contrast, peak concentrations for CK were observed at POST-24, whilst, differences between CON and CWI for all blood variables were unclear throughout the time points.

Total and individual Hooper scores were similar between conditions at PRE-EX. However, at POST-24, moderate to large differences between CON and CWI were noted for changes in total Hooper scores (*d* = 1.93), DOMS (*d* = 0.60), sensations of fatigue (*d* = 1.51), sleep quality (*d* = 1.36) and perceived stress (*d* = 1.56), with CWI having a positive effect on the exercise-mediated changes within these variables.

## Discussion

**FIGURE 3:**
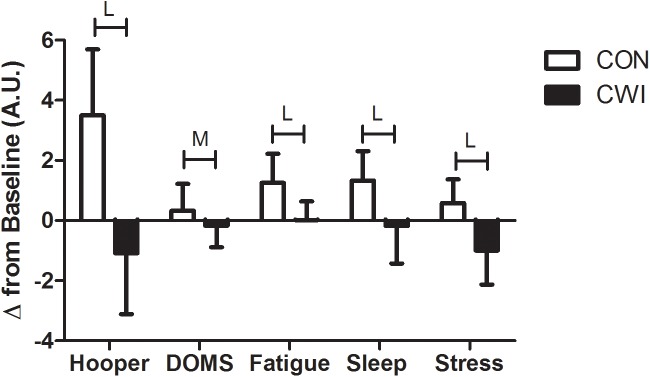
Changes in global Hooper score and specific scores for fatigue, stress, delayed onset muscle soreness (DOMS), and sleep, PRE-EX and 24-h following exercise (POST-24) according to recovery modalities: passive recovery (CON) or CWI. Between group differences are indicated by size of effect (S, small; M, moderate; L, large), and reported differences denote ≥75% likelihood that the stated effect is substantially different from the smallest worthwhile change (*d* = 0.2).

The main findings of the present study indicate that POST-R sprint (5- and 10-m) performances were ‘likely to very likely’ (Figures 1A,B, *d* = 0.64 and 0.65) impaired by prior CWI. However, moderate improvements in 10-m sprint performance were ‘likely’ evident at POST-24 after CWI compared with CON (Figure 1B, *d* = 0.53; -1.24 to 0.18). Additionally, the use of CWI ‘almost certainly’ resulted in a large overall improvement in Wellness/Hooper scores (Figure 3, *d* = 1.93; -2.60 to -1.25). Specifically, CWI ‘almost certainly’ resulted in improved sleep quality (*d* = 1.36; -2.04 to -0.68), stress (*d* = 1.56; -2.24 to -0.88) and perceived fatigue (*d* = 1.51; -2.19 to -0.84), and ‘likely’ resulted in a moderate decrease in DOMS (*d* = 0.60; -1.27 to 0.08). These findings as such render CWI as a suitable recovery intervention following MMA combat, particularly where the restoration of physical performance is required within a 24-h period. Within CON, small to moderate decreases in 10-m sprint, as well as CMJ and SJ performances were observed following (POST-24) simulated MMA combat (Figure 1). Such observations are in agreement with previous studies where sustained decrements in explosive, neuro-muscular performances were observed following a MMA training session (Lindsay et al., 2017) and simulated MMA competition (Ghoul et al., 2017). This decrement in muscular performance may be in-part explained by activity-induced muscle damage, likely sustained due to the collision, as well as time-sustained eccentric and/or isometric contractions during grappling moves, locks and holds performed during MMA activity. For instance, within rugby codes, both the severity of muscle damage, and the decrease in lower body neuro-muscular performances measured just after games/training sessions, have been shown to be associated to the intensity and frequency of collisions. (Tavares et al., 2017).

While a recent study (Lindsay et al., 2017) focused on the influence of CWI on the inflammatory/oxidative stress response following MMA combat, the recovery in physical performance has yet to be characterized. Accordingly, this is the first study to investigate the influence of CWI on the recovery of physical performance up to 24-h following simulated MMA combat. The present findings demonstrate that CWI enhanced the recovery of 10-m sprint performance at 24-h post simulated combat compared with CON (Figure 1). However, it is pertinent to note that 5-m sprint, as well as both jump performances were not influenced by CWI when compared to CON (Figure 1). It is likely that these performance measures were too brief and/or their changes influenced by CWI were too small to be detected. Alternatively, muscle recovery mediated by CWI might have little influence on the initial rate of force development (which are likely greater determinants of 5-m and jump performances) compared with extended force development, which would be better related to 10-m sprint performance.

Within the current study, it would be reasonable to consider that CWI might have improved the recovery of some performance measures (i.e., 10-m sprint) through reducing the extent of muscle damage and/or inflammation. While such reasoning would agree well with previous work documenting the beneficial effects of CWI on exercise-induced muscle damage (Pournot et al., 2010; Rowsell et al., 2011; Bleakley et al., 2012; Ihsan et al., 2016; Machado et al., 2016), the accompanying hematological data demonstrating no influence of CWI (Figure 2) clearly indicates an alternative mechanism by which sprint performance was improved. In the present study, the improved performance could potentially be explained by the reduced sensation of DOMS (Figure 3), independent of muscle damage. Indeed, perception of DOMS seems to be important to the recovery of exercise performance, as muscle pain independent of muscle damage has been shown to impair force generating capacity (Graven-Nielsen et al., 2002) Accordingly, CWI has been suggested to modulate the sensation of DOMS, and by extension muscle function, through limiting oedema (Ihsan et al., 2013; Choo et al., 2016) and/or through activating cutaneous receptors that mediate analgesia (Knowlton et al., 2013; Ihsan et al., 2016). Counterintuitive to the notion of recovery, CWI resulted in moderate and large decrements in POST-R for 5-m and 10-m sprint performances, respectively (Figure 1). Indeed, low intramuscular temperatures have been purported to result in delayed action potential generation, cross-bridge cycling and/or impaired fiber contractile properties. Moreover, the dynamic contractile force of the leg extensors has been shown to decrease by 4–6 % for every 1°C reduction in intramuscular temperatures (Bergh and Ekblom, 1979) However, the authors feel that these detrimental effects are likely transient in nature, and would likely recede following the rewarming of the musculature. Nevertheless, practitioners should proceed with caution when utilizing CWI between sprint/power based performance bouts scheduled close to each other.

The data also demonstrates an overall improvement in wellness measures 24 h following CWI (Figure 3), which could also in-part account for the improved recovery in sprint performance. This has been supported by recent findings that have demonstrated strong temporal associations between changes in wellness with the recovery of neuromuscular function and match running performances within team-sport athletes (Johnston et al., 2013; Ihsan et al., 2017). However, we acknowledge that the mechanisms underpinning improvements in post-exercise wellness following CWI are not entirely understood. It is suggested that improvements in perceptual recovery following CWI is modulated through changes in cardiac parasympathetic activity. For instance, enhanced parasympathetic activity following post-exercise CWI has been shown to be associated with reduced muscle soreness and perception of fatigue, as well as improved sense of relaxation (Stanley et al., 2012; Stanley et al., 2013; Choo et al., 2018) Additionally, perceptual recovery following CWI may also be related to alterations within the cerebral dopaminergic and noradrenergic systems, which have been implicated in the development of fatigue and may potentially be ameliorated following exposure to a cooling stimulus (Mundel et al., 2007; Roelands et al., 2013; Ihsan et al., 2016). However, the Hooper wellness battery also included ratings of sleep quality, which was seemingly improved following the use of CWI (Figure 3). This finding is somewhat in contrast with Robey et al. (2013), who demonstrated no influence of CWI on salivary melatonin, polysomnography or actigraphy-derived sleep measures. The reasons for such discrepancies are unclear, but perhaps there may be some dissociation between objective and subjective sleep measures, at least following the use of CWI use.

Although the simulated MMA combat protocol resulted in increased CK within both conditions at POST-24, there was no effect of CWI on CK concentration in the bloodstream (Figure 2). This finding is in agreement with some reports (Corbett et al., 2011; Pointon et al., 2011) but not all, as several investigations have reported decreased blood CK following CWI over a 24- to 72-h period (Eston and Peters, 1999; Ascensao et al., 2011; Minett et al., 2013). Such discrepancies within the literature may be due to differences in sampling time, nature of exercise and level of athletes. It was also suggested that CWI might facilitate physiological and perceptual recovery through altering the hormonal milieu. The current findings do not support this suggestion, as no changes in cortisol, testosterone or cortisol-to-testosterone ratio were observed following CWI (Figure 2). The present study findings are similar to Halson et al. (2008), who found no changes in cortisol or testosterone concentrations in response to post-exercise CWI. However, in the current study, we have characterized these hormonal changes for over a 24-h recovery period to elucidate potential mechanisms if any, as opposed to a 40-min post-exercise window undertaken in the study of Halson et al. (2008).

The present findings render CWI modality as a suitable recovery intervention during tournament scenarios or during intensive training camps/periods where athletes are required to compete regularly and the recovery/restoration of physical ability is paramount. The current data also advocates caution with regards to utilizing this recovery modality when performance bouts are closely scheduled together. In such scenarios, a warm-up exercises to facilitate the re-warming of the musculature are certainly warranted, and further studies should investigate this particular point.

We acknowledge several limitations associated with this study. Firstly, we were unable to monitor recovery exceeding 24 h, which could have been valuable in understanding the effects of CWI on longer term recovery. However, given that our participants were well-trained athletes, a 24 h recovery period is reasonable, considering their general ability to recover quicker, as well as their availability to commit to controlled experimental studies. Moreover, the lack of sport-specific performance variables could be considered a limitation as well. However, the choice of sprints and jumps, although general, are better controlled for, easily replicable and enables better objectivity when comparing with other studies.

## Conclusion

In summary, the present study examined the influence of post-exercise CWI on the recovery of physical performance, hematological stress markers and perceived wellness following a simulated MMA combat match. The use of CWI resulted in an enhanced recovery of 10-m sprint performance, as well as improved perceived wellness 24 h following simulated MMA combat. However, sprint/power ability was impaired when undertaken shortly after CWI. As such, active warm-ups are certainly warranted if CWI is administered in between performance bouts that are closely scheduled together. Hematological stress markers were not influenced by CWI, indicating that the physical and perceptual recovery was dissociated from the hormonal markers examined.

## Author Contributions

MT, NG, JC, and CT conceived and planned the experiments. MT, NG, JC, HC, and CT carried out the experiments. MT, MI, NG, JC, AC, CT, and KC contributed to the interpretation of the results. MT took the lead in writing the manuscript. All authors provided critical feedback and helped shape the research, analysis and manuscript.

## Conflict of Interest Statement

The authors declare that the research was conducted in the absence of any commercial or financial relationships that could be construed as a potential conflict of interest.
